# Role of miR200b-5p miRNA in lymphomagenesis associated with Sjögren’s syndrome (SS)

**DOI:** 10.31138/mjr.29.1.56

**Published:** 2018-03-19

**Authors:** Efstathia K. Kapsogeorgou, Athanasios G. Tzioufas

**Affiliations:** Department of Pathophysiology, School of Medicine, National and Kapodistrian University of Athens, Greece

**Keywords:** Sjögren’s Syndrome, non-Hodgkin’s lymphoma, miR200b-5p miRNA, minor salivary glands, lymphomagenesis

## Abstract

**Background:**

Development of non-Hodgkin’s lymphoma (NHL) is the major adverse outcome of primary Sjögren’s Syndrome (pSS) affecting both morbidity and mortality. The high frequency of transformation to lymphoid malignancy in pSS among autoimmune rheumatic diseases (6–10% of patients) and the accessibility of the affected organ (minor salivary glands; MSG), render pSS an ideal model for the study of lymphomagenesis associated with autoimmune diseases and inflammation. Although pSS-related lymphoid transformation is generally considered as an antigen-driven, multi-step process owed to the chronic activation of B-cells in MSGs, the underlying mechanisms remain elusive. Our recent results support that miR200b-5p miRNA is significantly down-regulated in the MSGs of pSS patients who have or will develop lymphoma, long before lymphoma clinical onset, indicating that it may be involved in lymphomagenesis.

**Aim:**

To investigate the role of miR200b-5p miRNA in pSS-associated lymphomagenesis.

**Methods:**

At first, the miR200b-5p-expression will be examined by *in situ* hybridization in MSGs of pSS patients who are at low risk and have not developed NHL during follow-up, high risk and developed NHL in the future (pre-lymphoma) or have NHL, and the expressing cellular types, as well as those with reduced expression during lymphomagenesis, will be identified. Then, the miR200b-5p targeted molecular pathways in those cellular types (epithelial, B-cells and/or other lymphocytes, all non-neoplastic) will be studied in *in vitro* experiments by over-expressing and silencing of miR200b-5p, followed by transcriptome analysis. This approach is expected to find possibly novel pathogenetic mechanisms underlying SS-related lymphomagenesis. The latter is of high significance, not only for the understanding of lymphomagenesis, but also for its reversal and/or treatment.

**Anticipated Benefits:**

This approach is anticipated to a) reveal the differentially regulated molecules and pathways by miR200b-5p, b) enlighten novel pathogenetic pathways underlying lymphomagenesis and c) identify novel therapeutic targets and possibly evidence-based therapeutic interventions.

## BACKGROUND/INTRODUCTION

The development of B-cell non-Hodgkin lymphoma (NHL) is common in primary Sjögren’s syndrome (pSS) (6–10% of patients) and affects both morbidity and mortality.^1–6^ In the majority of pSS patients, NHL starts in the exocrine glands, such as the salivary glands, which are the major target of pSS autoimmune responses. The high frequency of transformation to lymphoid malignancy in pSS among autoimmune rheumatic diseases^7,8^ and the accessibility of the affected organ, the minor salivary glands (MSG), render pSS an ideal model for the study of lymphomagenesis associated with autoimmune diseases and inflammation. The mechanisms underlying the development of neoplasia in pSS remain elusive. The existence of several clinical, laboratory and histological features present at diagnosis, including salivary gland enlargement (SGE), purpura, vasculitis, leukopenia, cryoglobulinemia, hypocomplementemia, rheumatoid factor, the severity of histopathologic MSG infiltrates, their organization into germinal centers (GC) and the infiltration by certain cell types, such as macrophages, in the pSS patients who are at high-risk to develop NHL in the future^3,9–15^ suggest that it is a chronic, multi-step process. Generally, it is considered that lymphomagenesis in pSS is a multistep process that arises from the chronic, continuous, antigen-driven B-cell stimulation resulting in the ineffective control of IgV gene recombination, chromosomal translocations, activation of proto-oncogenes, inactivation of tumor-suppressor genes, and ultimately to malignant transformation.^14,16^ The cellular source of the antigenic stimulation of B cells and the type of antigens are elusive. The organization of MSG infiltrates in ectopic germinal centers (GC) is considered critical for the activation of autoreactive B cells and the development of MALT lymphomas.^15,17,18^ Furthermore, salivary gland epithelial cells (SGECs) that are the key regulators of the pSS autoimmune responses in MSGs,^19,20^ have been shown to drive the differentiation of B cells, in a similar manner to that observed in MSG lesions,^19,21^ suggesting that they may be involved in the chronic activation of B cells and lymphomagenesis. The molecular pathways underlying the lymphomagenesis in pSS are under study.

Our recent evidence supports that miR200b-5p may be implicated in the lymphomagenesis associated with pSS. Although not de-regulated compared to sicca-controls, the expression levels of miR200b-5p in the MSGs were significantly reduced in pSS patients who were going to develop or had NHL compared to those who were at low-risk for developing lymphoma and did not had lymphoma during follow-up (^22^ *and unpublished data*). This decrease was evident long before the clinical onset of lymphoma, whereas the study of paired sequential MSG specimens before and on lymphoma diagnosis showed that the low miR200b-5p levels remained rather stable through transformation to lymphoma. These findings support that miR200b-5p may have a pathogenetic role in pSS-associated lymphomagenesis. Little is known for miR200b-5p, possibly because it represents the star strand, which is generally considered to degrade during miRNA biogenesis. Recently, it has been reported that miR200b-5p controls the non-canonical EMT in synergy with miR200b-3p by targeting PRKCA and PIP4K2A molecules in the RHOGDI pathway.^23^ However, the miR200b miRNAs, mainly miR200b-3p, are thought central regulators of oncogenesis, tumor metastasis and drug resistance of solid tumors by regulating epithelial-to-mesenchymal transition (EMT) primarily by down-regulation of the zinc-finger E-box-binding homeobox-1 (ZEB1) transcription factor and AKT activation.^24,25^ Furthermore, elevated miR200b expression and subsequent inhibition of ZEB1 transcription factor and increased BCL6 protein expression has been associated with better prognosis of the Helicobacter pylori-positive gastric diffuse large B-cell lymphomas, compared to Helicobacter pylori-negative ones.^26^

## AIM OF THE STUDY

Our findings support the implication of miR200b-5p miRNA in the lymphomagenesis in pSS. In this study, we propose the dissection of its role by identifying the expressing cells and the regulated molecular pathways.

## RESEARCH PLAN – METHODS

The cells expressing miR200b-5p in the MSG tissues, as well as those with reduced expression in MSGs from pre-lymphoma and lymphoma pSS patients, will be identified by *in situ* hybridization (ISH) using commercially available LNA-probes (Exiqon). Subsequently, the function of miR200b-5p in these cell types (SGEC, B, T and/or other type of lymphocytes) will be interrogated by silencing and overexpression *in vitro* experiments using specific miRNA inhibitors and mimics, respectively, followed by transcriptome analysis for the identification of the regulated genes and pathways. Finally, the pathogenetic potential of the identified pathways in the lymphomagenesis of pSS will be verified by investigating the expression of the molecules comprising the pathway in MSG tissues, SGECs and B cells from pSS patients with lymphoma or not. The expression of these molecules will be performed at the mRNA level by qPCR and at the protein level by immunohistochemistry in MSG tissues and/or immunoblotting and flow cytometry in cultured non-neoplastic cells (SGECs, B, T and/or other type of lymphocytes isolated from the peripheral blood of healthy donors).

The proposed study has been approved by the Ethics Committee of School of Medicine, NKUA, Greece (Protocol-No.: 1516023881).

## IMPACT OF THE STUDY

The study is expected to a) reveal the differentially regulated molecules and pathways by miR200b-5p, b) enlighten novel pathogenetic pathways with therapeutic potential and c) facilitate the better understanding of the mechanisms underlying lymphomagenesis in pSS, which is mandatory for the discovery of novel therapeutic targets and/or the evidence-based therapeutic administration of existing agents. The study of the pathogenetic mechanisms underlying progression to lymphoma in pSS is mandatory, since it may facilitate the arrest of the progression to and/or the effective treatment of malignancy in pSS, as well as other lymphoid malignancies, as suggested by the low MSG expression of miR200b-5p in a patient with HBV-sialadenitis and MALT lymphoma (*our unpublished data*).
